# Patient and Family Caregiver's Neuroticism and Conscientiousness Personality in Relation to Quality of Life of Patient With Parkinson's Disease: A Cross-Sectional Study Neuroticism and Conscientiousness Personality in Relation to QoL of Patient With PD

**DOI:** 10.3389/fneur.2018.00754

**Published:** 2018-09-11

**Authors:** Xiaoye Ma, Guilin Meng, Yan Tan, Xiaohui Liu, Yichen Zhao, Jia Yu, Aiping Jin, Yanxin Zhao, Xueyuan Liu

**Affiliations:** Department of Neurology, Shanghai Tenth People's Hospital, Tongji University School of Medicine, Shanghai, China

**Keywords:** conscientiousness, caregiver, neuroticism, personality, quality of life

## Abstract

**Background:** Personality impacts life expectancy and comprehensive treatment efficacy for patients with Parkinson's disease (PD). However, current research fails to involve the family caregiver's personality despite significant external support provided by family caregivers. This study explored neuroticism and conscientiousness personality factors of the patient and family caregiver associated with quality of life (QoL) of PD patients.

**Methods:** 134 couples of patients presenting with PD and their family caregivers that met inclusion criteria, were recruited for this cross-sectional study at Shanghai Tenth People's Hospital from October 2015 to 2017. The Parkinson's Disease Questionnaire-39 Items (PDQ-39) for QoL, including the physical component summary (PCS) and mental component summary (MCS), the Neuroticism Extraversion Openness Five-Factor Inventory (NEO-FFI) for neuroticism and conscientiousness personalities, and the Unified Parkinson's Disease Rating Scale (UPDRS) for PD severity questionnaires were employed. Multivariate stepwise linear regression determined the contributions of demographic, clinical and personality variables in PDQ-39, PCS, and MCS.

**Results:** PD patients and neuroticism were significantly associated with total PDQ-39, PCS, and MCS. Additionally, conscientiousness was significantly associated with PDQ-39 and PCS. After adding neuroticism and caregiver conscientiousness personality, the importance of neuroticism for the QoL model dramatically decreased, and caregiver conscientiousness personality was associated with lower scores in total PDQ-39 and PCS.

**Conclusion:** We revealed a significant association between neuroticism and physical or mental status of PD patients; however, this association decreased when caregiver conscientiousness was added to the model. Moreover, conscientiousness of patients and caregivers were associated with improved QoL.

## Introduction

Parkinson's disease (PD) is the second most common neurodegenerative in the world that is predominantly characterized by clinical manifestations that include bradykinesia, rigidity, tremor, and postural instability. PD poses a significant burden on the patient's family, not least due to associated costly therapy, but more importantly because PD represents a chronic disease that imparts a serious negative impact on the quality of life (QoL) of the affected patient (1).

The concept of QoL is a comprehensive manifestation of personal well-being, which impacts not only the life expectancy of the patient but also affects the efficacy of comprehensive treatment strategies. Considerable research has focused on how QoL is affected by a variety of physical and psychological factors that include the patient's age, level of education, disease duration, motor function impairment, cognitive deficit, and non-motor syndromes among others (2–4). Moreover, the aforementioned physical and psychological factors, have provoked increased interest in the potential role of personality, and an assessment of this clinical variable should be determined in the context of its likely contribution to QoL in PD (5). Personality represents an aggregated manifestation of a stable and characteristic pattern of temperament, cognition, emotion, and actions that are inherently unique to the individual in response to particular environmental situations (6), the dimensions of which, are extrovert behavior, neuroticism, conscientiousness, openness, and agreeableness—all of which represent the well-known five-factor model (5).

However, only a few studies have paid any particular attention to the association between personality and QoL in PD. Dubayova et al. reported that neuroticism might not only coincide with, but also precede the clinically-recognized motor onset seen in PD, and its impact on the QoL in PD patients (7). In Dubayova's subsequent studies, it was demonstrated that the personality of an individual that was influenced by poorly effective coping strategies, was negatively associated with the overall QoL in PD (8, 9). Further evidence that high neuroticism negatively affected QoL, while high conscientiousness (among other factors) were positive factors, was suggested in a relatively recent study (10). Besides the personality impacts of the patient on QoL in PD, a number of studies have addressed the impact of caregiver-burden, which has a variety persistent stress and impaired psychosocial functioning consequences that challenge caregiver QoL if not well adapted to the incidence of PD (11). In addition, so long as the interaction on informal family caregivers because of the PD, this interaction sent out from caregivers can be projected to patients as well. Thus, the personality of caregivers is also a factor that should be considered in the context of the PD patient's overall QoL. However, no study has thus far ever assessed the overall impact of the caregiver's personality on the QoL of PD patients.

Therefore, an improved understanding of the complex interactions of the patient, the family caregiver's personality and the associated QoL in PD is urgently required. Based on previous evidence of contradictory dominating effects of neuroticism and conscientiousness, we conducted the first survey of the impact of neurotic and conscientious personalities of the patient and family caregiver on the subsequent QoL of patients presenting with PD. In consideration of a high number of informal caregivers, we only investigated the care provided at home, including speaking to the patient's spouse, and their children or intimate relatives, while excluding the professional population of clinical caregivers at the hospital and clinic.

## Materials and methods

### Study design

This study was approved by the local ethics committee of Shanghai Tenth People's Hospital. In this cross-sectional study, patients with PD were recruited from the movement disorder neurology clinic between October 2015 and 2017 at the Shanghai Tenth People's Hospital affiliated to Tongji University. The interview invitation was sent when the patient visited the movement disorder neurology clinic. Under conditions where the patient and his/her family caregiver agreed to attend the study, the interview was immediately conducted during that visit. Caregivers included any close relative that lived with the patient, whether he/she was a spouse, adult, child, or intimate relative. Patients were assessed by a specialist in PD.

### Inclusion criteria

Patients that met all of the following inclusion criteria were eligible for the study:

1. Patients that adequately understood the purpose of the study, agreed to participate and signed the informed files.
2. Patients that were diagnosed with idiopathic PD according to the United Kingdom Parkinson's Disease Society Brain Clinical Criteria (12).
3. Patients that were older than 40 and less than 80 years of age.
4. Patients that had stable anti-Parkinsonian medication for at least 2 weeks.
5. Patients that were at the stage “on.”
6. Patients that had a stable company of at least one family caregiver.

### Exclusion criteria

1. A Mini-Mental State Examination (MMSE) score <24 points (13, 14).
2. Presence of severe infections, cardiopulmonary, cancer, liver or kidney disease or evidence of multisystem functional failure.
3. Presence of mental disorders or serious cognitive dysfunction or history of mental problems and abnormal behavior. During the interview, the National Institute of Neurological Disorders and Stroke-National Institute of Mental Health (NINDS-NIMH) (15) for psychosis in PD were applied and a diagnosis of PD-associated dementia was assigned according to MDS criteria (16) by 2 neurologists (Jia Yu and Yichen Zhao) with experience in movement disorders and dementias.
4. All the caregivers did not have a history of mental problems and abnormal behavior.

### QoL assessment of PD patients

The QoL of patients with PD was assessed using the Parkinson's Disease Questionnaire−39 Items (PDQ-39) (17), which is a disease-specific scale that was developed for measuring QoL in PD patients. The 39 items in this scale are divided into 8 sub-scales and include the following: mobility (10 items), activities of daily living (6 items), emotional well being (6 items), stigma (4 items), social support (3 items), cognition (4 items), communication (3 items), and bodily discomfort (3 items). These 8 subscales are further combined into 2 parts: Part 1: physical component summary (PCS) score (19 items, containing subscales of mobility, activities of daily living, and bodily discomfort), and Part 2: mental component summary (MCS) score (20 items, containing emotional well-being, stigma, social support, cognition and communication). In response to each question, patients select from answers scored from the following sliding scale: “never” (0), “occasionally” (1), “sometimes” (2), “often” (3) and “always” (4). The higher the score, the worse the QoL. The internal consistency of PDQ-39 was estimated as 0.85.

### Disease severity assessment of PD patients

Disease severity was measured by the Unified Parkinson's Disease Rating Scale (UPDRS) (18). UPDRS is used as a standard reference scale in clinical practice and in research for assessing disease severity in PD patients. Ratings are observation-based, and scores are obtained by interview and physical examination. The scale consists of four parts: mentation and mood (part 1), activities of daily living (part 2), motor function (part 3) and complications that result from dopaminergic therapy, including motor fluctuations and dyskinesia (part 4). Parts 1, 2, and 4 are interview-based, while part 3 is based on a clinical examination by a health professional, which represents the patient's condition at examination time. Patients can score from 0 to 176, with higher scores indicating increased disease severity.

### Neuroticism and conscientiousness assessment of PD patients and family caregivers

Extraversion Openness Five-Factor Inventory (NEO-FFI) (5) includes 60 projects, where 12 questions relate to each personality domain. Two of the 12 questions on each personality dimension are measured using a reversed scoring system. Every project is scored from the following criteria: “disagree very much” (1), “disagree to some degree” (2), “no emotional tendencies” (3), “agree to some degree” (4), and “agree very much” (5), with the highest score indicating a trend for that personality. We arranged a total of 24 questions with regard neuroticism and conscientiousness components from the NEO-FFI questionnaire. The results used in our study showed acceptably good reliability and validity with regard to the Chinese version, and reliability of the internal consistency of neuroticism and conscientiousness were estimated as 0.81 and 0.77.

### Statistical analysis

All statistical analyses were performed with the SPSS version 20.0 statistical software program (IBM, Armonk, NY, USA). The Kolmogorov-Smirnov test was used to investigate the normality of distribution. After the Kolmogorov-Smirnov test, all measurement data were in a normal distribution. Patient characteristics of the study population are presented as descriptive statistics [means (*SD*) or N (%)]. In the context of the PDQ-39 total score, and then with PCS and MCS as the outcome (dependent) variables, multivariate stepwise linear regression analysis with a forward selection of demographic, clinical and personality scores was used as independent variables across three models. The first model included gender, age, disease duration, and the UPDRS score. In the second model, patient neuroticism and conscientiousness were added. The third model also contained variables such as neuroticism and conscientiousness of the caregivers, which was based on model 2. One-way analysis of variance (ANOVA) was used to determine statistical significance of F values for the three models. Level of significance was set at an alpha value of *p* = 0.05.

## Results

### Descriptive statistics

**Figure 1:**
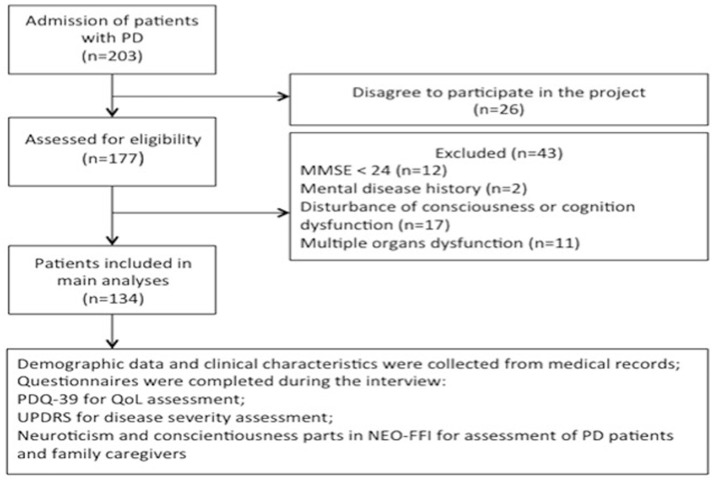
Flow chart of study implementation. PD, Parkinson's disease; MMSE, Mini-Mental State Examination; PDQ-39, Parkinson's Disease Questionnaire−39 Items; QoL, quality of life; UPDRS, Unified Parkinson's Disease Rating Scale; NEO-FFI, Extraversion Openness Five-Factor Inventory.

Out of 203 invited PD patients in the movement disorder neurology clinic at Shanghai Tenth People's Hospital, 177 patients recruited between October and October 2017, agreed to participate and filled in the questionnaires. However, 43 patients were excluded after the personal interview because of the conditions set out in the exclusion criteria. The sample thus consisted of 134 patients (response rate 66.0 %; Figure 1).

There were no differences seen between non-respondents and participants regarding gender, disease duration, and clinical course of PD. We checked the integrity of the questionnaire data for each patient and the corresponding caregiver in case of missing data. All PD patients took stable anti-parkinsonian therapy according to published international guidelines. Among the 134 patients with PD, the average age was 65.3 years (range 54–79 years); 53.7% of whom were males. The mean duration of PD was 8.4 years (*SD* = 5.0), and the UPDRS total score was 46.9 (*SD* = 18.4), meaning that the majority of patients were not in the early stages of disease progression. When analyzing PDQ-39, besides the total score, we combined the sections for “mobility,” “activities of daily living” and “bodily discomfort” (i.e., 19 items) into the PCS score. The remaining sections were “emotional well-being,” “stigma,” “social support,” “cognition,” and “communication” that were also aggregated together (i.e., 20 items) into the MCS score. From Table 1 it can be seen that among the average PDQ-39 total 428 score (mean = 50.3, *SD* = 16.7), the PCS was 24.2 (*SD* = 7.9), 429 which was lower than the MCS (mean = 26.1, *SD* = 9.9).

**Table 1:** Characteristics of the sample on study variables: PD subjects (*n* = 134).

**Demographic measures**	**Mean**	***SD***
Gender (male/female)	72/62	–
Age (years)	65.3	7.8
**CLINICAL MEASURES**		
Duration of PD (years)	8.4	5.0
UPDRS total score	46.9	18.4
**QUALITY OF LIFE MEASURES**		
PDQ-39 total score	50.3	16.7
PCS	24.2	7.9
MCS	26.1	9.9
**PERSONALITY OF PATIENTS**		
Neuroticism	20.9	8.4
Conscientiousness	27.8	7.3
**PERSONALITY OF CAREGIVERS**		
Neuroticism	18.2	6.2
Conscientiousness	28.7	6.9

*PDQ-39, Parkinson's Disease Questionnaire-39 Items; PCS, mental component summary of PDQ-39; MCS, mental component summary of PDQ-39; UPDRS, Unified Parkinson's Disease Rating Scale; PD, Parkinson's Disease; SD, standard deviation*.

### Multivariate stepwise linear analyses

Three models were constructed to explore the contribution to the variables of total PDQ-39, PCS, and MCS. In model 1, which consisted of patient age, gender, disease duration, and UPDRS score, the higher UPDRS was associated with a higher score in the PDQ-39 total score (beta = 0.44) including PCS and MCS data (beta = 0.36, and beta = 0.50, respectively) in PD patients. In addition, longer disease duration (beta = 0.23) was associated with a higher PCS score, and older age of the patient (beta = 0.21) was associated with a higher total PDQ-39 score.

When neuroticism and conscientiousness of the patients were included (i.e., model 2), the predictive strength of the model increased for PDQ-39 (Δadjusted *R*^2^ = 0.14), PCS (Δadjusted *R*^2^ = 0.12) and MCS (Δadjusted *R*^2^ = 0.11) as compared to model 1. Aside from UPDRS, which remained dominantly associated with three domains, the age of the patient remained the significant variable for the total PDQ-39 score. Moreover, neuroticism was significantly associated with a higher PDQ-39 score (beta = 0.27), PCS (beta = 0.22), and MCS (beta = 0.24), and conscientiousness was associated with a lower PCS score (beta = −0.20).

When neuroticism and conscientiousness of the caregiver were continued and added (i.e., model 3), then UPDRS remained significantly associated in general PDQ-39, PCS, and MCS, which means that a worse score for disease severity can lead to a worse QoL state. In addition, the neuroticism of the caregiver was not significantly related to the three domains of QoL, while conscientiousness of the caregiver was added to the predictor of PDQ-39 (beta = −0.22) and PCA (beta = −0.24). Compared to model 2, the partial correlation of patient neuroticism decreased 0.05 in the domain of MCS. Overall, this combination of variables in model 3 predicted 84.0% (adjusted *R*^2^) of the variance in general QoL (*p* < 0.01).

From Table 2 it can be seen that the adjusted coefficient of determination (adjusted *R*^2^) was increased with the introduction of independent variables in three models of the stepwise regression, which means that the predictive strength of the model increased from model 1 to model 3. For the three models of the stepwise regression analysis, we can see that all three models are statistically significant. Moreover, model 3 is the optimal regression model.

**Table 2:** Standard Coefficient (beta) in multiple regression analyses of 3 models.

	**Model 1**			**Model 2**			**Model 3**		
	**PDQ-39**	**PCS**	**MCS**	**PDQ-39**	**PCS**	**MCS**	**PDQ-39**	**PCS**	**MCS**
Age	0.21^*^	0.17	0.19	0.20^*^	0.18	0.12	0.20^*^	0.09	0.11
Gender	0.01	0.04	0.09	0.01	0.08	0.04	0.03	0.01	0.07
Duration of PD	0.18	0.23^*^	0.17	0.15	0.22^*^	0.13	0.12	0.22^*^	0.14
UPDRS	0.44^**^	0.36^**^	0.50^**^	0.40^**^	0.37^*^	0.46^*^	0.44^**^	0.35^*^	0.44^**^
P-neuroticism				0.27^**^	0.22^**^	0.24^**^	0.20^*^	0.22^*^	0.20^*^
P-conscientiousness				−0.03	−0.20^*^	−0.07	−0.06	−0.32	−0.21
C-neuroticism							0.03	0.06	0.05
C-conscientiousness							−0.22^**^	−0.11	−0.24^**^
Adjusted *R*^2^	0.36	0.35	0.26	0.50^*^	0.47	0.37^*^	0.54^**^	0.55	0.48^*^

Standardized Coefficients (beta) values above are significant at

* P < 0.05 (2-tailed), and

** *P < 0.01 (2-tailed)*.

*PDQ-39, Parkinson's Disease Questionnaire-39 Items; PCS, physical component summary of PDQ-39; MCS, mental component summary of PDQ-39; UPDRS, Unified Parkinson's Disease Rating Scale; P-neuroticism, neuroticism personality of patient; P-conscientiousness, conscientiousness personality of patient; C-neuroticism, neuroticism personality of caregivers; C-conscientiousness, conscientiousness personality of caregiver; PD, Parkinson's Disease*.

## Discussion

Our study has demonstrated a significant association between neuroticism and the physical and mental status of PD patients, where regression analysis showed that disease severity was the strongest predictor of impaired QoL and that personality traits played a complementary role in QoL.

As expected, most predictors of QoL deterioration reflect demographic and baseline disease characteristics, such as older age and longer duration. Many of these factors have been previously found to be associated with poor QoL in patients with PD (19, 20).

Furthermore, we revealed a significant association between neuroticism and physical or mental status of PD patients. In previously published studies, the association between personality and QoL was explored in PD patients. Positive coping personality was both positively associated with QoL and inversely correlated with the UPDRS (21–23). Similar results were found in other neurodegenerative diseases like Alzheimer's disease (AD) (24) or multiple sclerosis (MS) (25).

Moreover, conscientiousness of patients and caregivers were associated with improved QoL. the association between patient neuroticism with QoL decreased when faced with the corresponding caregiver's high level of consciousness—although there was no doubt that its importance in the context of QoL is important and plays a role. Conscientiousness of both the patient and the caregiver are associated with an improved QoL. As in the NEO-FFI model, neuroticism represents a tendency to be sensitive, sad, guilty, depressed, anger-prone, and nervous, and as such, easily delivers complex and unpleasant emotions in such a way that is unreasonable. By contrast, conscientiousness refers to a dutiful, organized, self-disciplinary characteristic, which is usually always associated with a relatively strict expectation of a set standard of behavioral expectations. A possible reason for this might be that the interaction between the patient and the caregiver implies that when the patient was largely influenced by a neurotic personality, the caregiver's duty and disciplinary predilection could help the patient cope with an attacking episode of PD.

Coping with illness is not a solitary process in a family. When one is affected by a PD attack, the family is also affected. Furthermore, family caregivers often take responsibility for providing physical and emotional support to PD patients that have a limited capacity to participate in regular social activities.

The prevalence of PD increases with age and this number will double by the year 2030 (26, 27). Since currently available drugs do not control disease progression, patient compliance and satisfaction are subsequently depressingly low (28). In addition, the current management of PD is limited to symptomatic improvement of troublesome symptoms of the disease, and there has always emphasized the measurement of quality of life as an outcome metric that measures the impact of disease on patients' lives (29). Our findings suggest that identified issues in patient and family caregiver's neuroticism and conscientious personality as it related to the QoL of the PD patient should be explored, which can have significant health-related, social, and economic implications, and not only for patients and caregivers but also for the healthcare system.

## Strengths and limitations

The study's main strength is its integration of the patient and caregiver's personality in the study analytics. To our knowledge, a new point of view has been revealed, which could be helpful in advancing understanding of the potential factors and complexities of QoL.

One of the limitations of this study is the recognition that this is a cross-sectional study and does not take into account longitudinal changes in the QoL. Moreover, due to a lack of a gold standard for evaluating the effects of scoring neuroticism and consciousness, the models we have designed must be confirmed by conducting large-sample studies.

## Conclusions

In conclusion, the integration of the patient and caregiver's personality could be more important than the patient's personality in assessing QoL, which might lead to the provision of a higher quality care and improved functional recovery of the patient presenting with PD. These might lead to the provision of higher quality care and better functional recovery and community support to re-integrate the patient suffering from PD.

## Data sharing and data accessibility

All data are available without restriction from the corresponding author on reasonable request.

## Author contributions

XuL and YaZ conceived and designed the study; GM, YT, XiL, YiZ, JY, and AJ performed data analysis; XM wrote the paper.

### Conflict of interest statement

The authors declare that the research was conducted in the absence of any commercial or financial relationships that could be construed as a potential conflict of interest.

## References

[1] Tabert MH Manly JJ Liu X Pelton GH Rosenblum S Jacobs M. Neuropsychological prediction of conversion to Alzheimer disease in patients with mild cognitive impairment. Arch Gen Psychiatry (2006) 63:916–24. 10.1001/archpsyc.63.8.916 16894068

[2] D'Iorio A Vitale C Piscopo F Baiano C Falanga AP Longo K. Impact of anxiety, apathy and reduced functional autonomy on perceived quality of life in Parkinson's disease. Parkinson Related Disord. (2017) 43:114–7. 10.1016/j.parkreldis.2017.08.003 28797564

[3] Yamamoto T Uchiyama T Higuchi Y Asahina M Hirano S Yamanaka Y. Long term follow-up on quality of life and its relationship to motor and cognitive functions in Parkinson's disease after deep brain stimulation. J Neurol Sci. (2017) 379:18–21. 10.1016/j.jns.2017.05.037 28716237

[4] Tu XJ Hwang WJ Ma HI Chang LH Hsu SP. Determinants of generic and specific health-related quality of life in patients with Parkinson's disease. PloS ONE (2017) 12:e0178896. 10.1371/journal.pone.0178896 28650957 PMC5484474

[5] Poletti M Bonuccelli U. Personality traits in patients with Parkinson's disease. assessment and clinical implications. J Neurol. (2012) 259:1029–38. 10.1007/s00415-011-6302-8 22083431

[6] Santangelo G Piscopo F Barone P Vitale C. Personality in Parkinson's disease: clinical, behavioural and cognitive correlates. J Neurol Sci. (2017) 374:17–25. 10.1016/j.jns.2017.01.013 28087060

[7] Dubayova T Nagyova I Havlikova E Rosenberger J Gdovinova Z Middel B. Neuroticism and extraversion in association with quality of life in patients with Parkinson's disease. Qual Life Res. (2009) 18:33–42. 10.1007/s11136-008-9410-x 18989757

[8] Dubayova T Nagyova I Havlikova E Rosenberger J Gdovinova Z Middel B. The association of type D personality with quality of life in patients with Parkinson's disease. Aging Mental Health (2009) 13:905–12. 10.1080/13607860903046529 19888711

[9] Dubayova T Krokavcova M Nagyova I Rosenberger J Gdovinova Z Middel B. Type D, anxiety and depression in association with quality of life in patients with Parkinson's disease and patients with multiple sclerosis. Qual Life Res. (2013) 22:1353–60. 10.1007/s11136-012-0257-9 23054489

[10] Pontone GM Mari Z Perepezko K Weiss HD Bassett SS. Personality and reported quality of life in Parkinson's disease. Int J Geriat Psychiatry (2017) 32:324–30. 10.1002/gps.4475 27059809 PMC5333497

[11] Greenwell K Gray WK van Wersch A van Schaik P Walker R. Predictors of the psychosocial impact of being a carer of people living with Parkinson's disease: a systematic review. Parkinson Related Disord. (2015) 21:1–11. 10.1016/j.parkreldis.2014.10.013 25457815

[12] Fahn S Elton RL Members of the updrs development committee unified Parkinson's disease rating scale in recent developments in Parkinson's disease. N J Macmill Health Inform. (1987) 2:153–63.

[13] Folstein MF Folstein SE McHugh PR“Mini-mental state”. A practical method for grading the cognitive state of patients for the clinician. J Psychiatr Res. (1975) 12:189–98. 1202204 10.1016/0022-3956(75)90026-6

[14] Foltynie T Brayne CE Robbins TW Barker RA. The cognitive ability of an incident cohort of Parkinson's patients in the UK. The CamPaIGN study. Brain (2004) 127 (Pt 3):550–60. 10.1093/brain/awh067 14691062

[15] Ravina B Marder K Fernandez HH Friedman JH McDonald W Murphy D. Diagnostic criteria for psychosis in Parkinson's disease: report of an NINDS, NIMH work group. Mov Disord. (2007) 22:1061–8. 10.1002/mds.21382 17266092

[16] Emre M Aarsland D Brown R. Clinical diagnostic criteria for dementia associated with Parkinson's disease. Mov Disord. (2007) 22:1689–707. 10.1002/mds.21507 17542011

[17] Peto V Jenkinson C Fitzpatrick R. PDQ-39: a review of the development, validation and application of a Parkinson's disease quality of life questionnaire and its associated measures. J Neurol. (1998) 245(Suppl. 1):S10–4. 10.1007/PL00007730 9617716

[18] Movement Disorder Society Task Force on Rating Scales for Parkinson's D. The Unified Parkinson's Disease Rating Scale (UPDRS): status and recommendations. Mov Disord. (2003) 18:738–50. 10.1002/mds.10473 12815652

[19] Schrag A Jahanshahi M Quinn N. What contributes to quality of life in patients with Parkinson's disease?. J Neurol Neurosurg Psychiatry (2000) 69:289. 10.1136/jnnp.69.3.308 10945804 PMC1737100

[20] Duncan GW Khoo TK Yarnall AJ O'Brien JT Coleman SY Brooks DJ. Health-related quality of life in early Parkinson's disease: the impact of nonmotor symptoms. Mov Disord. (2014) 29:195–202. 10.1002/mds.25664 24123307

[21] Gison A Dall'Armi V Donati V Rizza F Giaquinto S. Dispositional optimism, depression, disability and quality of life in Parkinson's disease. Funct Neurol. (2014) 29:113–19. 10.11138/FNeur/2014.29.2.113 25306121 PMC4198159

[22] Gison A Rizza F Bonassi S Dall'Armi V Lisi S Giaquinto S. The sense-of-coherence predicts health-related quality of life and emotional distress but not disability in Parkinson's disease. BMC Neurol. (2014) 14:193. 10.1186/s12883-014-0193-0 25304029 PMC4197289

[23] Gison A Rizza F Bonassi S Donati V Giaquinto S. Effects of dispositional optimism on quality of life, emotional distress and disability in Parkinson's disease outpatients under rehabilitation. Funct Neurol. (2015) 30:105–11. 10.11138/FNeur/2015.30.2.105 26415782 PMC4610757

[24] Gacek M. Selected personality - related determinants of alcohol beverage consumption among Polish elite team sport athletes. Rocz Panstw Zakl Hig. (2016) 67:263–9. 27546323

[25] Mohamadi A Davoodi-Makinejad M Azimi A Nafissi S. Personality characteristics in MS patients: the role of avoidant personality. Clin Neurol Neurosurg. (2016) 144:23–7. 10.1016/j.clineuro.2016.02.035 26963086

[26] Dorsey ER Constantinescu R Thompson JP Biglan KM Holloway RG Kieburtz K. Projected number of people with Parkinson disease in the most populous nations, 2005 through 2030. Neurology (2007) 68:384–6. 10.1212/01.wnl.0000247740.47667.03 17082464

[27] Luan K Joanne R Ahmed HA. Factors that lead to hospitalisation in patients with Parkinson disease—A systematic review. Int J Clin Pract. (2017) 72:e13039. 10.1111/ijcp.13039 29119656

[28] Tarazi FI Sahli ZT Wolny M Mousa SA. Emerging therapies for Parkinson's disease: from bench to bedside. Pharmocol Ther. (2014) 144:123–33. 10.1016/j.pharmthera.2014.05.010 24854598

[29] Wagner J Ball J. Implications of the institute of medicine report: evaluation of biomarkers and surrogate endpoints in chronic disease. Clin Pharmacol Therapeut. (2015) 98:12–5. 10.1002/cpt.129 25833004

